# Descriptive Profile of Upper Limb Injuries in a Specialized Police Intervention Unit: A Retrospective Study

**DOI:** 10.7759/cureus.108381

**Published:** 2026-05-06

**Authors:** Diogo Rodrigues, Nuno Domingues, Tiago Dias Domingues, Pedro Barradas

**Affiliations:** 1 Physical Medicine and Rehabilitation, São José Local Health Unit, Lisbon, PRT; 2 Center for Statistics and Applications, Faculty of Sciences, University of Lisbon, Lisbon, PRT; 3 Mathematical Sciences, Faculty of Sciences, University of Lisbon, Lisbon, PRT; 4 Orthopedics, Portuguese Public Security Police, Lisbon, PRT

**Keywords:** descriptive study, law enforcement, musculoskeletal injury, police intervention unit, upper limb injury, work incapacity

## Abstract

Background: Police work is associated with high physical demands and an increased risk of occupational injury, particularly among specialized tactical units exposed to intense operational activity. Musculoskeletal injuries are common in law enforcement personnel and frequently result in work incapacity, but data specifically addressing upper limb injuries in intervention units remain limited. The aim of this study was to characterize the clinical profile of upper limb injuries reported as duty-related accidents in a Portuguese police intervention unit and to analyze their association with surgical treatment and the duration of work incapacity.

Methods: A retrospective observational study was conducted, including all upper limb injury events reported between January 1, 2021, and December 31, 2025, in a specialized unit of the Portuguese Public Security Police Intervention Corps. Data were obtained from the official injury registry. Variables included age, police rank, anatomical location, injury presentation, diagnosis category, need for surgical treatment, and duration of temporary total work incapacity. Analyses were performed at the event level. Comparisons were performed using Welch’s t-test, the exact Wilcoxon-Mann-Whitney test, and Kendall’s τ_b_ correlation coefficient.

Results: A total of 54 injury events were analyzed, of which 27 (50.0%) resulted in temporary total work incapacity. The shoulder was the most frequently affected site, with 15 (27.8%) injuries, followed by fingers with 12 (22.2%) and hand with nine (16.7%). Superficial soft-tissue injuries were the most common diagnosis, occurring in 18 (33.3%), followed by fractures in 10 (18.5%). Surgical treatment was required in eight (14.8%) events and was associated with longer incapacity duration compared with conservative treatment (Z = -3.88, p < 0.01). The median duration of incapacity among events with sick leave was 90 days (31-151). No significant association was found between age and sick leave or duration of incapacity (p = 0.285 and 0.267, respectively). Injuries involving the arm and shoulder showed the longest incapacity periods.

Conclusions: Upper limb injuries in this specialized police unit frequently resulted in work incapacity, with surgical treatment and proximal upper limb injuries associated with longer recovery time. These findings highlight the burden of musculoskeletal injuries in tactical law enforcement personnel and emphasize the need for further research to better inform prevention and rehabilitation strategies.

## Introduction

Police work is widely recognized as a physically demanding and hazardous occupation, exposing officers to a higher risk of injury than most civilian professions. Law enforcement personnel are frequently required to perform strenuous tasks such as running, restraining suspects, carrying heavy loads, and operating in unpredictable and potentially violent environments, all of which increase the likelihood of occupational injury [1-3]. Epidemiological studies have consistently shown that musculoskeletal injuries account for the majority of work-related injuries in police populations and represent a major cause of lost workdays, long-term disability, and organizational burden [4,5].

The risk of injury among police officers is influenced by the wide range of duties performed, including operational activities, physical training, and routine occupational tasks. Although assaults and high-risk interventions are often perceived as the main causes of injury, a substantial proportion of injuries occur during nonviolent activities such as manual handling, training, falls, or repetitive physical tasks [1,6]. Surveillance studies based on administrative and compensation databases have reported injury rates in law enforcement personnel that are higher than those observed in most other occupations, highlighting the need for detailed characterization of injuries in this population [4].

Musculoskeletal disorders are particularly prevalent among police officers, with strains, sprains, and soft-tissue injuries being the most commonly reported diagnoses [5,7]. Several studies have identified the shoulder, back, and upper extremity as frequent injury sites, reflecting the physical demands associated with defensive tactics, weapon handling, load carriage, and physical control techniques [7,8]. Investigations conducted in specialized police units have suggested that injury patterns may differ from those observed in general-duty officers, likely due to higher physical loads, the use of tactical equipment, and more frequent exposure to high-intensity situations [6,9].

Training environments also represent an important source of injury. Studies involving police recruits and tactical trainees have reported high injury incidence during academy training and physical conditioning programs, with musculoskeletal injuries occurring in a substantial proportion of participants [10-12]. Similar findings have been described in military and other tactical populations, in which repetitive load, intense physical activity, and operational training contribute to a high prevalence of both overuse and traumatic injuries [13,14]. These observations suggest that injury patterns in tactical professions share common characteristics across different organizations, emphasizing the importance of injury surveillance and prevention strategies.

Beyond injury occurrence, the consequences of occupational trauma in law enforcement often include prolonged work incapacity, need for surgical treatment, and long-term functional limitations. Previous studies have shown that injury severity, anatomical location, and type of tissue damage are important determinants of recovery time and return to duty, with upper limb injuries frequently associated with significant work restrictions because of their impact on manual function [5,7]. Despite the relevance of these outcomes, there is limited literature specifically addressing upper limb injuries in police officers, particularly in operational units exposed to high physical demands.

The Portuguese Public Security Police Intervention Corps is a specialized tactical unit responsible for high-risk operations, including riot control, tactical intervention, and public order missions. These duties involve intense physical effort, frequent use of force, and the use of protective equipment, which may predispose officers to injury patterns that differ from those observed in general-duty police populations. To date, no published studies have specifically analyzed upper limb injuries reported as duty-related accidents in this type of unit.

Therefore, the aim of this retrospective study was to characterize the profile of upper limb injuries reported as duty-related accidents in a Portuguese police intervention unit between 2021 and 2025, and to evaluate their anatomical distribution, injury presentation, need for surgical treatment, and association with duration of work incapacity.

## Materials and methods

Study design and data source

A retrospective observational study was conducted to evaluate upper limb injuries reported as duty-related accidents in a specialized unit of the Portuguese Public Security Police Intervention Corps between January 1, 2021, and December 31, 2025. Data were obtained from the official injury registry of the unit and corresponded to routinely collected administrative and medical records. Each record represented a single injury event, and no individual identifier was available; therefore, the unit of analysis was the injury event rather than the individual. All upper limb injury events reported as duty-related accidents during the study period were included, and no exclusion criteria were applied.

Variables and outcomes

Collected variables included police rank, age at the time of injury, year of occurrence, anatomical location, injury presentation, diagnosis code according to the International Classification of Diseases, 11th Revision (ICD-11), diagnostic description, need for surgical intervention, duration of temporary total work incapacity, and duration of partial work incapacity. The variable originally recorded as “injury mechanism” included both injury mechanisms and clinical presentations (e.g., pain-related or nonspecific complaints), as obtained from registry data, and is therefore interpreted descriptively as injury presentation.

Diagnoses were grouped into broader categories based on ICD-11 classification, including superficial soft-tissue injuries, tendinous pathology, tendon rupture, ligament or joint capsule injury, fractures, and nonspecific injuries. Sick leave was defined as any period of temporary total work incapacity lasting more than 0 days.

The main outcomes were anatomical distribution of injuries, injury category, need for surgical treatment, and duration of temporary total work incapacity. Additional analyses explored associations between age and sick leave, age and duration of incapacity, police rank and number of injury events, need for surgery and duration of incapacity, injury category and surgical treatment, and anatomical location and duration of incapacity. Because denominator population data were not available, incidence rates could not be calculated, and the analysis was limited to a descriptive event-based approach.

Statistical analysis

Age was summarized as mean ± standard deviation, and the remaining continuous variables as median (interquartile range), first quartile-third quartile (Q1-Q3). Categorical variables were summarized as absolute and relative frequencies. Age was compared between events with and without sick leave using Welch’s two-sample t-test. Duration of incapacity was compared between injuries requiring and not requiring surgical treatment using the exact Wilcoxon-Mann-Whitney test. The association between age and duration of incapacity was assessed using Kendall’s τ_b_. Statistical significance was defined as p < 0.05. Because individual identifiers and denominator population data were not available, independence between observations could not be verified, and inferential results were interpreted with caution. Analyses were performed using R software, version 4.5.1 (R Foundation for Statistical Computing, Vienna, Austria).

Ethical considerations

The study was authorized by the Training Directorate of the Portuguese Public Security Police. As this was a retrospective analysis of anonymized data, ethics committee approval and informed consent were not required.

## Results

A total of 54 upper limb injury events reported as duty-related accidents were registered during the study period. Because the registry did not contain an individual identifier, it was not possible to determine whether multiple events corresponded to the same officer; therefore, all analyses were performed at the event level.

The general characteristics of the injury events are summarized in Table 1. The mean age at the time of injury was 41.63 ± 8.1 years. Most events occurred among officers with the rank of Senior Police Officer, accounting for 34 (63.0%), followed by Police Officer with 16 (29.6%), while Sergeant and Lieutenant each accounted for 2 (3.7%). Because denominator population data were unavailable, the relationship between rank and injury occurrence could not be formally tested.

**Table 1:** Characteristics of upper limb injury events (n = 54) according to police rank Values are presented as n (%) for categorical variables and as mean ± SD or median (IQR), according to data distribution. Data are shown according to officer rank SD: standard deviation; IQR: interquartile range (Q1-Q3)

Variable	Police officer (n = 16)	Senior police officer (n = 34)	Sergeant (n = 2)	Lieutenant (n = 2)	Overall (n = 54)
Age (years)					
Mean ± SD	35.1 ± 4.4	45.03 ± 7.6	42 ± 5.7	36 ± 9.9	41.63 ± 8.1
Duration of temporary total work incapacity (days)					
Median (IQR)	0 (0-52)	8 (0-90)	107 (0-214)	15 (0-30)	1.5 (0-90)
Anatomical site					
Shoulder	5 (31.3%)	9 (26.5%)	1 (50.0%)	0 (0%)	15 (27.8%)
Arm	0 (0%)	3 (8.8%)	1 (50.0%)	1 (50.0%)	5 (9.3%)
Elbow	1 (6.3%)	3 (8.8%)	0 (0%)	0 (0%)	4 (7.4%)
Forearm	0 (0%)	1 (2.9%)	0 (0%)	0 (0%)	1 (1.9%)
Wrist	2 (12.5%)	5 (14.7%)	0 (0%)	1 (50.0%)	8 (14.8%)
Hand	5 (31.3%)	4 (11.8%)	0 (0%)	0 (0%)	9 (16.7%)
Fingers	3 (18.8%)	9 (26.5%)	0 (0%)	0 (0%)	12 (22.2%)
Injury presentation					
Contusion	3 (18.8%)	8 (23.5%)	1 (50.0%)	0 (0%)	12 (22.2%)
Sprain/strain	2 (12.5%)	6 (17.6%)	1 (50.0%)	0 (0%)	9 (16.7%)
Pain/nonspecific	5 (31.3%)	8 (23.5%)	0 (0%)	2 (100%)	15 (27.8%)
Fracture	5 (31.3%)	5 (14.7%)	0 (0%)	0 (0%)	10 (18.5%)
Superficial injury	0 (0%)	5 (14.7%)	0 (0%)	0 (0%)	5 (9.3%)
Dislocation	1 (6.3%)	2 (5.9%)	0 (0%)	0 (0%)	3 (5.6%)
Surgical intervention					
No	15 (93.8%)	28 (82.4%)	1 (50.0%)	2 (100%)	46 (85.2%)
Yes	1 (6.3%)	6 (17.6%)	1 (50.0%)	0 (0%)	8 (14.8%)
Injury category					
Superficial soft-tissue	3 (18.8%)	14 (41.2%)	1 (50.0%)	0 (0%)	18 (33.3%)
Fracture	5 (31.3%)	5 (14.7%)	0 (0%)	0 (0%)	10 (18.5%)
Tendinous pathology	3 (18.8%)	4 (11.8%)	0 (0%)	1 (50.0%)	8 (14.8%)
Tendon rupture	1 (6.3%)	5 (14.7%)	1 (50.0%)	0 (0%)	7 (13.0%)
Ligament/capsule	2 (12.5%)	4 (11.8%)	0 (0%)	1 (50.0%)	7 (13.0%)
Nonspecific	2 (12.5%)	2 (5.9%)	0 (0%)	0 (0%)	4 (7.4%)

Regarding anatomical distribution, the shoulder was the most frequently affected site, with 15 (27.8%), followed by the fingers with 12 (22.2%), the hand with nine (16.7%), and the wrist with eight (14.8%). Less frequent locations included the arm with five (9.3%), elbow with four (7.4%), and forearm with one (1.9%).

With respect to injury presentation, pain-related conditions or nonspecific complaints were the most frequently reported, accounting for 15 (27.8%), followed by contusions with 12 (22.2%), fractures with 10 (18.5%), sprains or strains with nine (16.7%), superficial injuries with five (9.3%), and dislocations with three (5.6%).

When diagnoses were grouped by injury category, superficial soft-tissue injuries were the most frequent, accounting for 18 (33.3%), followed by fractures with 10 (18.5%), tendinous pathology with eight (14.8%), tendon rupture with seven (13.0%), ligament or joint capsule injuries with seven (13.0%), and nonspecific injuries with four (7.4%).

Temporary total work incapacity occurred in 27 (50.0%) injury events, whereas the remaining 27 (50.0%) did not result in sick leave. Among the events with temporary total work incapacity, the median duration of leave was 90 days (31-151). The wide variability in duration was related to the presence of extreme values, including one event with more than 400 days of incapacity (Table 2).

**Table 2:** Characteristics of injury events according to sick leave Values are presented as n (%) for categorical variables and as mean ± SD or median (IQR), according to data distribution. Age was compared between the groups using Welch’s two-sample t-test. Other variables are presented descriptively due to the absence of denominator population data and small cell counts SD: standard deviation; IQR: interquartile range (Q1-Q3)

Variable	No sick leave (n = 27)	Sick leave (n = 27)	Overall (n = 54)	Test statistic	p value
Age (years)					
Mean ± SD	42.8 ± 7.8	40.4 ± 8.3	41.6 ± 8.1	t = 1.08	0.285
Temporary total work incapacity (days)					
Median (IQR)	-	90 (31-151)	1.5 (0-90)	-	-
Partial work incapacity (days)					
Median (IQR)	-	0 (0-0)	0 (0-0)	-	-
Rank					
Police Officer	9 (33.3%)	7 (25.9%)	16 (29.6%)	-	-
Senior Police Officer	16 (59.3%)	18 (66.7%)	34 (63.0%)		
Sergeant	1 (3.7%)	1 (3.7%)	2 (3.7%)		
Lieutenant	1 (3.7%)	1 (3.7%)	2 (3.7%)		
Anatomical site					
Shoulder	9 (33.3%)	6 (22.2%)	15 (27.8%)	-	-
Arm	1 (3.7%)	4 (14.8%)	5 (9.3%)		
Elbow	3 (11.1%)	1 (3.7%)	4 (7.4%)		
Forearm	1 (3.7%)	0 (0%)	1 (1.9%)		
Wrist	4 (14.8%)	4 (14.8%)	8 (14.8%)		
Hand	1 (3.7%)	8 (29.6%)	9 (16.7%)		
Fingers	8 (29.6%)	4 (14.8%)	12 (22.2%)		
Injury presentation					
Contusion	8 (29.6%)	4 (14.8%)	12 (22.2%)	-	-
Sprain/strain	3 (11.1%)	6 (22.2%)	9 (16.7%)		
Pain/nonspecific	13 (48.1%)	2 (7.4%)	15 (27.8%)		
Fracture	0 (0%)	10 (37.0%)	10 (18.5%)		
Superficial injury	2 (7.4%)	3 (11.1%)	5 (9.3%)		
Dislocation	1 (3.7%)	2 (7.4%)	3 (5.6%)		
Surgical intervention					
No	27 (100%)	19 (70.4%)	46 (85.2%)	-	-
Yes	0 (0%)	8 (29.6%)	8 (14.8%)		
Injury category					
Fracture	0 (0%)	10 (37.0%)	10 (18.5%)	-	-
Ligament/capsule	4 (14.8%)	3 (11.1%)	7 (13.0%)		
Nonspecific	4 (14.8%)	0 (0%)	4 (7.4%)		
Superficial soft-tissue	11 (40.7%)	7 (25.9%)	18 (33.3%)		
Tendinous pathology	7 (25.9%)	1 (3.7%)	8 (14.8%)		
Tendon rupture	1 (3.7%)	6 (22.2%)	7 (13.0%)		

A comparison of events with and without sick leave showed similar age distributions between the groups. The mean difference in age was 2.37 years (95% confidence interval (CI) = -2.93 to 6.77), and this difference was not statistically significant (Welch t-test: t = 1.08, df = 51.74, p = 0.285). No significant correlation was found between age and duration of incapacity (Kendall’s τ_b _= 0.15, p = 0.267).

Surgical treatment was required in eight (14.8%) events, whereas 46 (85.2%) were treated conservatively. All events requiring surgery resulted in sick leave. Among the events with temporary total work incapacity, those requiring surgical intervention had longer leave periods than those treated conservatively, and this difference was statistically significant (exact Wilcoxon-Mann-Whitney test: Z = -3.88, p < 0.01) (Table 3).

**Table 3:** Duration of temporary total work incapacity for events resulting in sick leave (n = 27) according to surgical intervention Values are presented as median (IQR). Differences in the duration of temporary total work incapacity between the groups were assessed using the exact Wilcoxon-Mann-Whitney test IQR: interquartile range (Q1-Q3)

Variable	No surgery (n = 19)	Surgery (n = 8)	Test statistic	p value
Median (IQR)	40 (14-90)	190 (141.5-255.5)	Z = -3.88	<0.01

The distribution of surgical intervention according to injury category is shown in Table 1. Tendon rupture was the injury category most frequently associated with surgery, accounting for four (50.0%) of the surgical cases. Ligament or joint capsule injuries and nonspecific injuries were not associated with surgical intervention.

Analysis according to anatomical site showed that injuries involving the hand resulted in sick leave in eight (88.9%) events, and arm injuries in four (80.0%). The longest periods of temporary total work incapacity were observed in arm injuries, with a median of 159.5 (79.5-252.0) days, followed by shoulder injuries with 78.5 (14.0-221.0) days and hand injuries with 90.0 (46.0-126.5) days. Wrist and finger injuries were associated with shorter incapacity periods. Although the shoulder was the most frequently affected anatomical site, most shoulder injuries, nine (60.0%), did not result in sick leave.

## Discussion

This retrospective study aimed to characterize the descriptive and clinical profile of upper limb injuries reported as duty-related accidents in a specialized police intervention unit over a five-year period and to analyze their association with surgical treatment and duration of work incapacity. The main findings were that half of the injury events resulted in temporary total work incapacity; surgical intervention was associated with longer periods of leave; tendon rupture was the injury type most frequently requiring surgery; and injuries involving the arm and shoulder were associated with the longest incapacity periods. Overall, the results highlight the burden of musculoskeletal injuries in police populations, particularly in units exposed to high physical demands.

The distribution of injuries observed in the present study is consistent with previous reports describing law enforcement as an occupation with high exposure to physical hazards, including physical confrontation, load carriage, and repetitive upper limb use [1,2]. In the current series, the shoulder, fingers, and hand were the most frequently affected anatomical regions, which agrees with prior studies showing that upper limb injuries are common in police officers, especially in tactical or intervention units [3,5,6]. Similar patterns have been reported in specialist policing populations, where upper extremity injuries often occur during training, defensive tactics, or operational activities requiring manual force or weapon handling [3,8].

Superficial soft-tissue injuries were the most frequent diagnostic category, followed by fractures and tendinous disorders. This distribution is comparable to that described in large occupational surveillance studies, in which contusions, sprains, and fractures account for a substantial proportion of police injuries [4,5]. However, although soft-tissue injuries were the most common, more severe injuries such as fractures and tendon ruptures were more likely to result in prolonged incapacity or surgical treatment, highlighting the difference between frequency and clinical impact.

Half of the injury events resulted in temporary total work incapacity, with a median duration of approximately three months among cases requiring leave. This finding is in line with previous epidemiological studies reporting that musculoskeletal injuries are a major cause of lost work time among law enforcement officers [4,5,7]. The wide variability in incapacity duration observed in the present study, including extreme values exceeding one year, has also been described in tactical and military populations, where injury severity and recovery time are highly heterogeneous [12-14].

No significant association was found between age and the occurrence of sick leave or the duration of incapacity. Although increasing age has been associated with higher injury risk or longer recovery time in some studies [7,11], other reports in police and military populations have shown inconsistent results, particularly in relatively homogeneous operational units where physical fitness requirements remain high across age groups [12,14]. The absence of a clear age effect in the present study may therefore reflect the specific characteristics of the studied unit.

Surgical intervention was required in a minority of cases but was strongly associated with longer periods of incapacity. All events requiring surgery resulted in sick leave, and the duration of incapacity was significantly greater than that for conservatively treated injuries. This finding is consistent with previous reports showing that injuries requiring operative management are among the main determinants of prolonged work absence in tactical occupations [5,12]. Tendon rupture was the injury category most frequently associated with surgery, which is consistent with the functional impact of these injuries and the frequent need for surgical repair.

Injuries involving the arm and shoulder were associated with the longest periods of temporary total work incapacity. Upper limb injuries affecting proximal segments may have a greater impact on functional capacity, particularly in occupations requiring manual strength, weapon handling, and physical confrontation. Similar observations have been reported in both police and military studies, where shoulder and upper limb injuries are often associated with longer rehabilitation periods and delayed return to duty [3,12,14]. Interestingly, although the shoulder was the most frequently affected anatomical site, most shoulder injuries did not result in sick leave, suggesting that many cases may correspond to minor or self-limited conditions.

The present study has several limitations that must be considered when interpreting the results. First, the analysis was performed at the event level because no individual identifier was available, and therefore, it was not possible to determine whether multiple injuries occurred in the same officer. Second, denominator population data were unavailable, which prevented calculation of injury incidence rates and limited the analysis to descriptive comparisons. These limitations have been recognized in previous occupational injury surveillance studies and restrict the ability to identify risk factors or causal relationships [4,6]. Third, the relatively small sample size reduced the possibility of performing inferential statistical tests for some comparisons, particularly when cell counts were low.

Despite these limitations, the study provides relevant information on the profile and consequences of upper limb injuries in a specialized police unit. The results highlight the importance of musculoskeletal injuries as a cause of work incapacity and emphasize the need for further research to better inform prevention and rehabilitation strategies in tactical law enforcement populations. Future studies, including individual-level data and denominator population information, are needed to better identify risk factors and guide targeted injury prevention programs.

## Conclusions

Upper limb injuries reported as duty-related accidents in this specialized police unit frequently resulted in temporary total work incapacity, with considerable variability in recovery time. More severe injuries, particularly fractures and tendon ruptures of upper extremities, were associated with surgical treatment and longer absence from duty, and injuries involving proximal upper limb segments showed the greatest impact on work incapacity. These findings highlight the occupational burden of upper limb injuries in tactical law enforcement personnel and support the need for further research to better inform prevention and rehabilitation strategies. Because the analysis was event-based and denominator population data were not available, the results should be interpreted as descriptive.
